# An Unusually Deep Corallimorph Barren on a Remote Coral Reef in the South‐Central Red Sea

**DOI:** 10.1002/ece3.73442

**Published:** 2026-04-09

**Authors:** Walter A. Rich, Viktor Nunes Peinemann, Nicole J. Burt, Darren J. Coker, Jess S. Glanz, Michael L. Berumen, Maggie D. Johnson, Michael D. Fox

**Affiliations:** ^1^ Marine Science Program, Biological and Environmental Sciences and Engineering Division King Abdullah University of Science and Technology Thuwal Saudi Arabia; ^2^ KAUST Coral Restoration Initiative (KCRI) King Abdullah University of Science and Technology Thuwal Saudi Arabia

**Keywords:** Corallimorph barren, Farasan banks, Phase shift, *Rhodactis rhodostoma*

## Abstract

Corallimorphs are opportunistic cnidarians that can compete with reef‐building corals for substrate and thrive in disturbed conditions. Their clonal reproduction often results in a patchy distribution, and they typically occupy a negligible percentage of benthic area on tropical coral reefs. In the Red Sea, the corallimorph *Rhodactis rhodostoma* has been observed in dense aggregations inhabiting shallow reef flats and nearshore habitats, and is less abundant on reef slopes deeper than 3 m. Here, we describe an unusually deep corallimorph barren on the exposed slope of a remote offshore reef 96 km from the Saudi Arabian coast in the south‐central Red Sea. Based on molecular and morphological characteristics, we tentatively identify the species as *R.* cf. *rhodostoma*. Mean percent cover of *R.* cf. *rhodostoma* ranged from 25% to 35% from depths of 15–30 m, while it was completely absent at 5 m. Although mean percent cover of *R*. cf. *rhodostoma* was less than 50% for all depths surveyed, localized patches resulted in areas consisting of ~90% coverage. Contrary to many reports of corallimorph barrens appearing after a physical disturbance, there are no obvious factors promoting corallimorph dominance on this remote reef. Further surveys are needed to document the total extent of the corallimorph barren and to assess if other reefs in the region are susceptible to similar phenomena.

## Graphical abstract

We report a corallimorph barren on a remote coral reef in the southern Red Sea. Unlike previous studies that show corallimorphs prefer shallow habitats in disturbed conditions, corallimorphs were absent in the shallow areas of the reef but dominated the substrate from 15 to at least 30 m depth. The cause of this dominance is unclear, as the uninhabited reef shows no obvious signs of local human impacts.

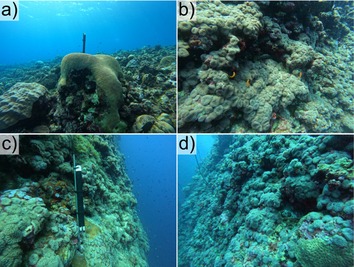

## 1 Introduction

Corallimorphs are clonal anthozoans that can compete with scleractinian corals for benthic space (Kuguru et al. 2004; Chadwick and Morrow 2011). Typically occurring in small, non‐contiguous aggregations (Chen et al. 1995), some corallimorph species can rapidly dominate benthic communities following disturbances through asexual clonal replication (Muhando et al. 2002; Solandt and Wood 2005; Work et al. 2008; Norström et al. 2009). When a previously rare taxa exceeds 25% of benthic cover, the new benthic state is considered a “barren” (Reimer et al. 2021), but corallimorph outbreaks can result in near 100% cover and prevent other species from re‐establishing due to their rapid clonal reproduction and potent allelopathic compounds (Miles 1991; Langmead and Chadwick‐Furman 1999; Carter et al. 2019). Although most commonly associated with a physical disturbance, like shipwrecks or nutrient pollution (Muhando et al. 2002; Work et al. 2008; Carter et al. 2019), corallimorph outbreaks have been increasingly reported across the Indo‐Pacific with uncertain causes (Crane et al. 2016; Tkachenko et al. 2022; Anto et al. 2023; Reimer et al. 2025).

In the Red Sea, the corallimorph *Rhodactis rhodostoma* is most commonly found on shallow coral reefs (< 3 m depth) in small aggregations (Fishelson 1970; Chadwick‐Furman and Spiegel 2000). Outside of these gregarious patches, *R. rhodostoma* accounts for a negligible component of benthic cover, especially in deeper habitats on reef slopes (Chadwick‐Furman and Spiegel 2000; Kuguru et al. 2007). Most studies on Red Sea *R. rhodostoma* have occurred in the Gulf of Aqaba, where a phase shift has occurred from a coral‐dominated to corallimorph‐dominated community on reef flats following an extreme low tide event (Fishelson 1973; Norström et al. 2009). *Rhodactis rhodostoma* exhibits a number of characteristics that allow it to thrive in shallow reef habitats, such as tolerance to high‐irradiance and high‐temperature conditions (Kuguru et al. 2007), high clonal growth rates (Chadwick‐Furman and Spiegel 2000), and antagonistic behavior against hard coral species (Langmead and Chadwick‐Furman 1999). It seems to prefer sheltered conditions, as they occur in higher abundance on inner reef flats compared to the exposed outer reef flat (Chadwick‐Furman and Spiegel 2000). Outside of these well‐studied sites in the Gulf of Aqaba, corallimorphs are rarely reported in benthic surveys in the wider Red Sea basin.

Here, we report an unusually deep observation of a corallimorph barren on the exposed reef slope of a remote offshore reef in the south‐central Red Sea. Contrary to previous reports of corallimorphs preferring shallow habitats (Chadwick‐Furman and Spiegel 2000; Muhando et al. 2002; Kuguru et al. 2004, 2007; Work et al. 2018), we observed a complete absence of individuals shallower than 5 m, but high densities of corallimorphs from depths of 15 m to at least 30 m.

## 2 Methods

**FIGURE 1:**
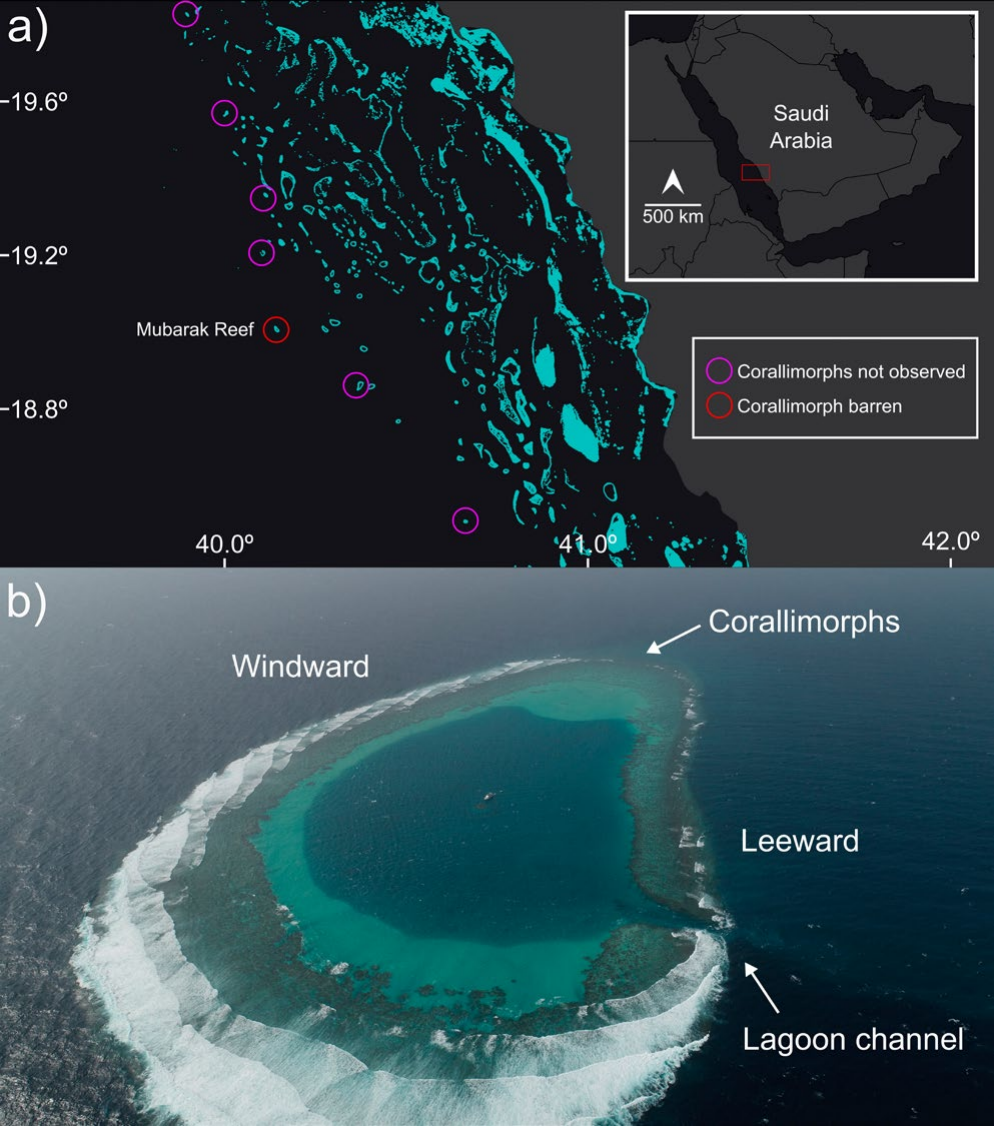
(a) Mubarak Reef (red circle) is part of the Farasan Banks reef system (light blue shading) along the Saudi Arabian coast in the south‐central Red Sea. Corallimorphs were not observed at six other reefs (magenta circles) during the expeditions when this study was carried out. (b) An aerial view of Mubarak Reef. The corallimorph barren was observed on the northern tip of the reef, roughly 1.5 km from the lagoon channel. This photo was captured on a day with strong southerly winds, but the prevailing winds are northwesterly, and the corallimorph barren is situated on the exposed side of the reef. Photo taken in February 2019 by A. Gusti.

We visited the northern tip of Mubarak Reef (19.014978°N, 40.138043°E) as part of a broader survey effort in the Farasan Banks region of the south‐central Red Sea during two expeditions in June and October 2024 (Figure 1a). Mubarak Reef is an atoll‐like tower reef typical of the outer Farasan Banks (Rowlands and Purkis 2015), and features a shallow lagoon with a single channel on the eastern edge of the reef (Figure 1b). The reef is situated 96 km from the Saudi Arabian coast on the shelf edge of the wider Farasan Banks reef system, ~60 km from the main shipping lane in the central Red Sea (Larayedh et al. 2024), and is surrounded by deep water. Due to its isolated position, Mubarak Reef experiences low fishing pressure and human impacts, as it is an impractical target for artisanal fishing which comprises the majority of fishing in the region (Kattan et al. 2017).

**FIGURE 2:**
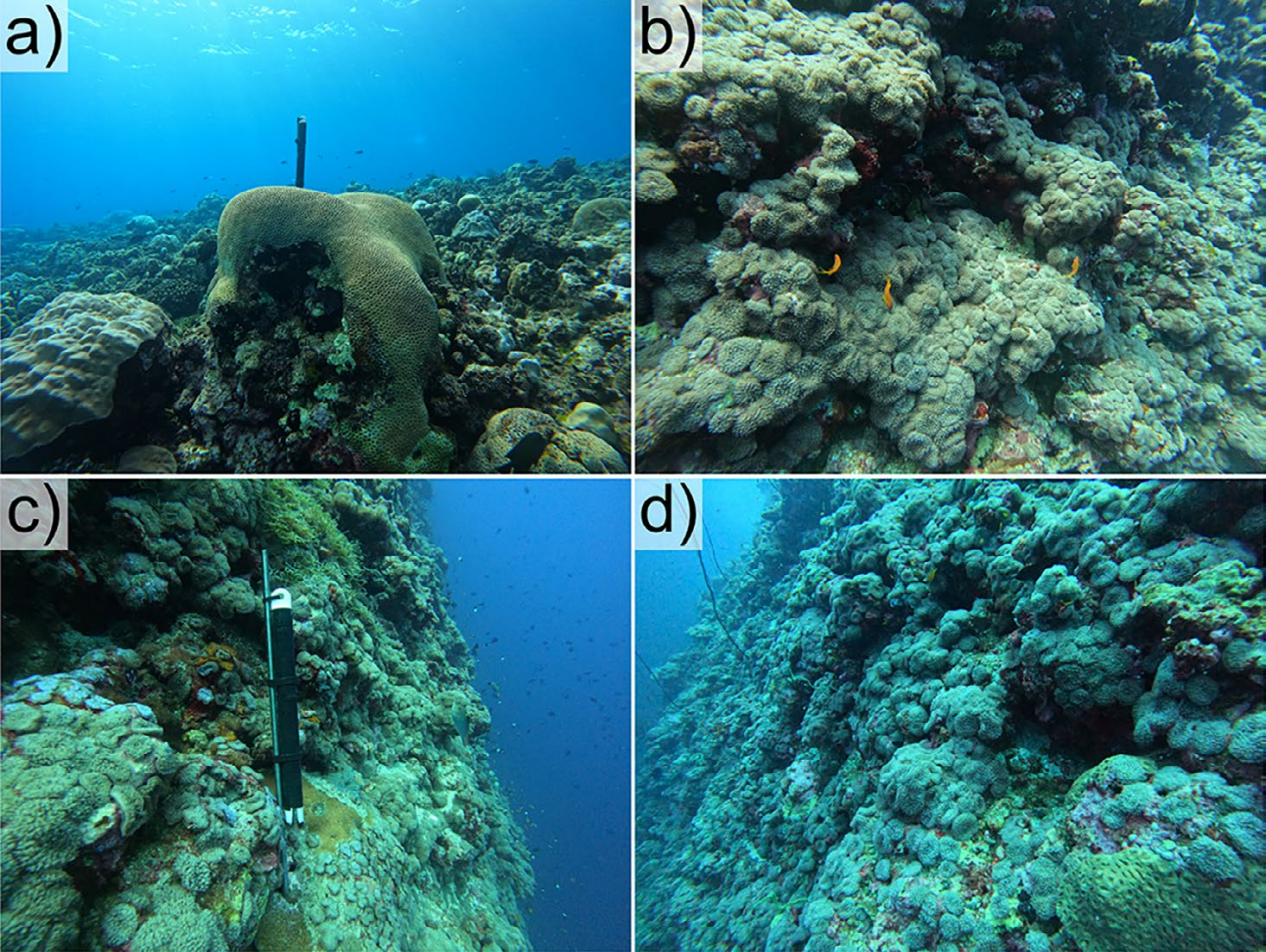
Corallimorphs (*Rhodactis* cf. *rhodostoma*) were not detected at 5 m (a), but they dominated the benthic community at 15 m (b), 20 m (c), and 30 m depth (d) along the northern slope of Mubarak Reef. Photos taken on 7 June 2024 by W.A. Rich.

During our two expeditions we visited seven offshore reefs and performed a total of 41 dives along the western edge of the Farasan Banks, and only encountered corallimorphs in abundance at Mubarak Reef (Figure 2 and Data S1). The five southernmost reefs (including Mubarak Reef) hosted similar coral communities, with massive and encrusting species comprising the majority of coral morphotypes, and few branching species represented. The two northernmost reefs boasted higher coral cover and had a larger proportion of branching species at all depths. To quantify corallimorph distribution at Mubarak Reef, we conducted depth‐stratified phototransects at 5, 15, 20, and 30 m depths. At each depth photos were taken every meter along a 30 m transect (an Olympus Stylus Tough GX‐7 camera positioned 1 m from the substrate) for a total of 31 photos per depth. The benthic area covered by each photo was approximately 0.66 m^2^, resulting in roughly 20 m^2^ of substrate surveyed at each depth. The transects were placed roughly vertically aligned with each other down the reef slope and we surveyed toward the southwest from the starting point. Photos were analyzed in CoralNet (coralnet.ucsd.edu) using 50 stratified random points per image. The area under each point was annotated by lowest identifiable taxa and assigned to one of six functional groups: corallimorphs; sand, rubble, or pavement; turf, macroalgae, or cyanobacteria; hard coral; crustose coralline algae (CCA); or other invertebrates. Percent cover was calculated based on functional group assignments. Analyses and figures were generated in R (R Core Team 2023), and a benthic shape file of the Farasan Banks region was sourced from Allen Coral Atlas (Allen Coral Atlas 2022).

Given the unique occurrence of this deep, high‐density corallimorph area, we collected specimens for genetic identification to confirm if the species was the common *R*. *rhodostoma*. We collected two specimens from different depths (25 m and 15 m) and froze them at −20°C on the liveaboard boat without a fixative. Unfortunately, given the opportunistic nature of the sampling, we did not have proper materials onboard to properly preserve a voucher specimen. Upon returning to the laboratory, the samples were transferred to a −80°C freezer until analysis. We used the Qiagen DNEasy Blood & Tissue Kit to extract genomic DNA from the sample. Before the final DNA elution, we incubated the DNA filters at 56°C for 5 min to improve the DNA yield. We then amplified a region of the cytochrome c oxidase subunit 1 (COI) gene using the primer set jgLCO1490 and jgHCO2198, with an annealing temperature of 48°C (Geller et al. 2013). A region of the 16S gene was also amplified using the primer sets Z16Sf and Z16Sr, with an annealing temperature of 60°C (Forsman et al. 2010). The resulting sequences were cleaned and aligned in Geneious Prime 2025.0.3, resulting in a 667 bp 16S sequence with 97% of basepairs above Q30 and a 402 bp COI sequence with 89% of basepairs above Q30. Both sequences were deposited in GenBank under accession numbers PV221264 (16S) and PV223401 (COI). Sequence similarity was assessed using the Basic Local Alignment Search Tool (BLAST) against the NCBI GenBank database.

## 3 Results and Discussion

The 16S sequence shares a 99.2% sequence identity with a sequence of *R. rhodostoma* provided by Cha (2007), which was from a specimen collected in Oman. The COI and 16S sequences of our samples also have > 99% sequences identity with *Rhodactis indosinensis* and *Amplexidiscus fenestrafer*. Due to the slow evolution of these genes in the clade (Romano and Palumbi 1997; Shearer et al. 2002), they are insufficient to separate certain closely related species. The former species, *R. indosinensis*, ranges from the Great Barrier Reef to Japan (Chen and Miller 1996), while the latter, *A. fenestrafer*, has digitiform discal tentacles (Cha 2007). As our observations are in the Red Sea, within the known distribution of *R. rhodostoma*, and our samples resemble that species morphologically, including branching discal tentacles, we tentatively identify it as *R. rhodostoma*.

**FIGURE 3:**
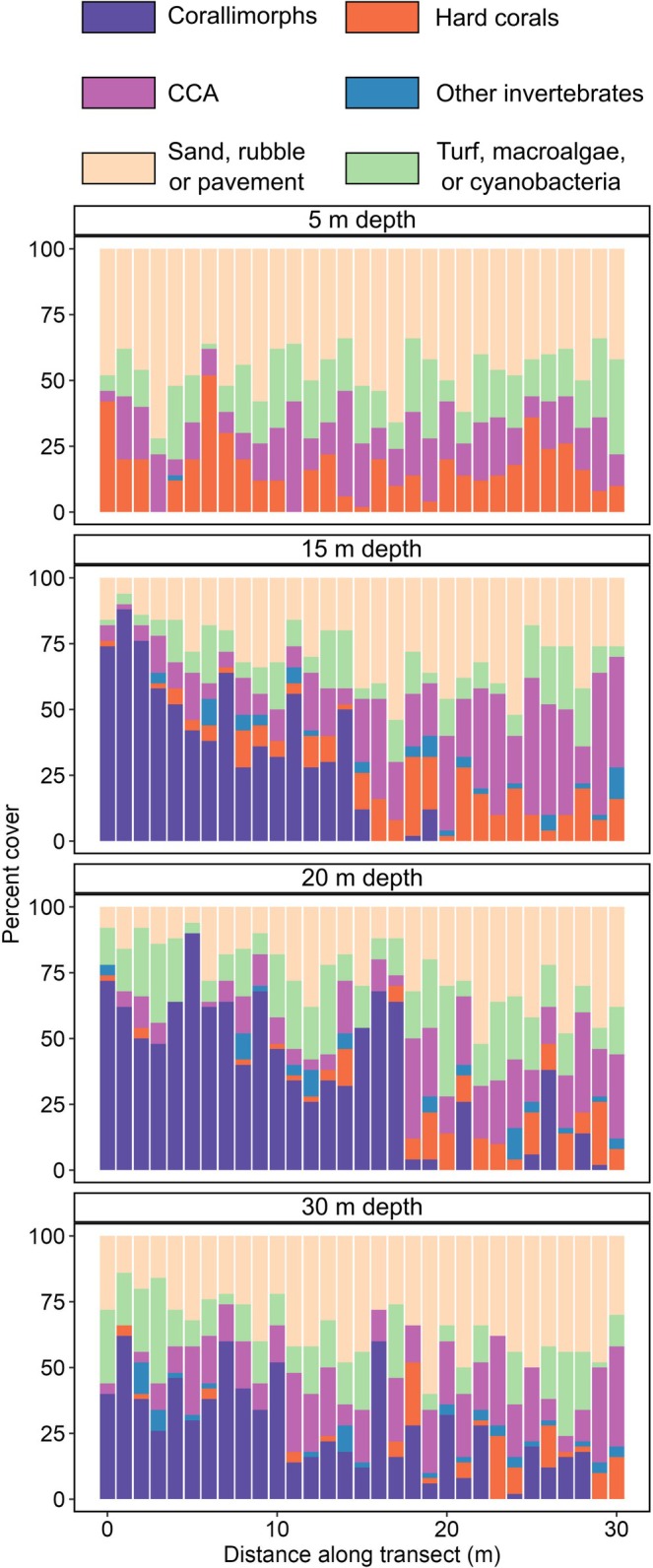
Benthic composition of phototransects for the four depths surveyed. Each bar depicts percent cover of benthic categories for one photo; photos were taken every meter along a 30 m transect. Percent cover of corallimorphs (*Rhodactis* cf. *rhodostoma*) was 0 ± 0, 25.1 ± 4.8, 34.6 ± 5.8, and 25.7% ± 6.1% (mean ± standard error) for 5, 15, 20, and 30 m depths, respectively.

Along the reef slope, corallimorphs comprised between 0% and 88% of benthic area among individual photos, with a mean of 25.1%, 34.6%, and 25.7% at 15, 20, and 30 m, respectively (Figure 3). Corallimorphs were absent along the 5 m transects. While we did not collect data deeper than 30 m, the high‐density cover of corallimorphs extended past our visual range, well beyond 30 m depth (*pers. obs*.). Our observations run contrary to the typical patterns observed in other areas of the Red Sea and wider Indo‐Pacific, where corallimorphs are more abundant in shallow, high‐light areas—particularly reef flats and coastal sublittoral habitats—and rapidly decrease in abundance with depth (Chadwick‐Furman and Spiegel 2000; Muhando et al. 2002; Kuguru et al. 2004, 2007; Work et al. 2018). The mean percent cover we observed at 15–30 m depths is similar to what has been reported for *R. rhodostoma* on shallow reef flats in Tanzania (15%–28%) (Kuguru et al. 2004), whereas that same study reported only 3% cover at the deepest site surveyed (6 m). In the northern Red Sea at 18 m depth, mean density of *R. rhodostoma* was only 2 individuals m^−2^, compared to 15 and 11 individuals m^−2^ at 0.5 and 3 m depths, respectively (Kuguru et al. 2007). At the site of a shipwreck in Palmyra Atoll, density of another corallimorph species (*R. howesii*) reached 288 polyps m^‐2^ (Work et al. 2008), but percent cover was highest at 5 m and declined with depth; they were not detected at the deepest survey depth of 21 m (Work et al. 2018). The lack of corallimorphs at shallower depths in our survey does not appear to be due to competition with other organisms, as bare substrate made up 48% of the 5 m transect (Figure 3). Other physical factors, like high wave action, may have prevented the barren from expanding to shallower depths.

The mean percent cover ranging from 25% to 35% for transects ≥ 15 m depth obscures the localized dominance of corallimorphs due to the patchy nature of the barren. In many individual photos, corallimorphs comprised the majority of the photo area, while corallimorphs were completely absent in other photos within the same transects (Figure 3). For example, at 15 m depth, the first half of the 30 m transect contained corallimorphs in every photo with an overall mean of 50.1% cover, and the final 15 m was comprised mostly of bare substrate or CCA. We may have surveyed the southwestern edge of the barren, as corallimorphs extended along the northeast of the reef wall for another ~50 m (D.J. Coker, *pers. obs*., Data S1). Our survey effort is therefore likely an underestimate of the true density and spatial extent of this unique benthic community. Nevertheless, corallimorphs were clearly dominant compared to hard corals they typically compete with for space: corallimorph percent cover was 2.5, 5.5, and 5.7‐fold higher than hard coral percent cover at 15, 20, and 30 m, respectively.

Corallimorph barrens are often associated with physical disturbance, such as shipwrecks (Work et al. 2008; Carter et al. 2019), low tide events (Norström et al. 2009), or chronic nutrient pollution (Muhando et al. 2002). But Mubarak Reef is located far offshore and has few human visitors, and there are no reports of shipwrecks on this reef. The corallimorph barren is located ~1.5 km from the lagoon channel on the windward side of the reef (Figure 1b), and we did not observe a corallimorph barren on two dives near the channel entrance, so flushing of lagoon water likely did not cause the conditions which facilitated corallimorph growth. This localized area of corallimorph dominance is unlikely linked to local human impacts and is consistent with recent reports of inexplicable corallimorph outbreaks in Vietnam, India, and Japan (Tkachenko et al. 2022; Anto et al. 2023; Reimer et al. 2025).

Widespread coral mortality associated with ocean warming and increasingly severe marine heatwaves may facilitate climate‐driven opportunities for corallimorph expansion in the absence of direct local human stressors. The Farasan Banks region experienced major impacts from the 2015/2016 coral bleaching event, with drastic declines in coral cover (Berumen et al. 2019; DeCarlo et al. 2020; Gonzalez et al. 2024). It is possible that extensive coral mortality could have reduced competition for fast‐growing corallimorphs that were already established at Mubarak Reef, which may have spread rapidly over newly free substrate. However, the other six reefs we surveyed in the area did not host corallimorph barrens despite suffering similar heat stress. In general, coral reefs in the Farasan Banks are less well studied compared to other regions in the Red Sea (Cochran et al. 2024), and we are unaware of previous surveys at this exact site on Mubarak Reef. With limited historical data, we cannot identify how long this barren has persisted or the primary driver for this anomaly in benthic community dominance. Future monitoring is needed to track potential expansion of *R*. cf. *rhodostoma* throughout Mubarak Reef, as established corallimorph barrens can persist for years to decades (Norström et al. 2009; Work et al. 2018; Carter et al. 2019). Continued corallimorph dominance will likely limit recovery potential of coral communities, particularly as climate‐related stressors such as marine heatwaves are likely to occur more frequently (Oliver et al. 2018).

## Author Contributions

**Walter A. Rich:** conceptualization (lead), data curation (lead), formal analysis (lead), investigation (lead), methodology (lead), visualization (lead), writing – original draft (lead), writing – review and editing (equal). **Viktor Nunes Peinemann:** formal analysis (equal), writing – review and editing (equal). **Nicole J. Burt:** data curation (supporting), writing – review and editing (equal). **Darren J. Coker:** data curation (supporting), writing – review and editing (equal). **Jess S. Glanz:** data curation (supporting), writing – review and editing (equal). **Michael L. Berumen:** resources (equal), supervision (equal), writing – review and editing (equal). **Maggie D. Johnson:** resources (equal), supervision (equal), writing – review and editing (equal). **Michael D. Fox:** resources (lead), supervision (lead), writing – review and editing (equal).

## Conflicts of Interest

The authors declare no conflicts of interest.

## Supporting information


**Video S1:** ece373442‐sup‐0001‐VideoS1.mp4.

## Data Availability

Data is available from the Dryad data repository: DOI: https://doi.org/10.5061/dryad.pg4f4qs3t.
